# An intracellular, non-oxidative factor activates in vitro chromatin fragmentation in pig sperm

**DOI:** 10.1186/s40659-023-00467-w

**Published:** 2023-10-24

**Authors:** Estel Viñolas-Vergés, Marc Yeste, Ferran Garriga, Sergi Bonet, Yentel Mateo-Otero, Jordi Ribas-Maynou

**Affiliations:** 1 Biotechnology of Animal and Human Reproduction (TechnoSperm), Institute of Food and Agricultural Technology, University of Girona, S17003 Girona, Spain; 2 Unit of Cell Biology, Department of Biology, Faculty of Sciences, University of Girona, S17003 Girona, Spain; 3 Catalan Institution for Research and Advanced Studies (ICREA), S08010 Barcelona, Spain

**Keywords:** DNA, Ejaculated sperm, Fragmentation, Pig, Toroid-linker regions

## Abstract

**Background:**

In vitro incubation of epididymal and vas deferens sperm with Mn^2+^ induces Sperm Chromatin Fragmentation (SCF), a mechanism that causes double-stranded breaks in toroid-linker regions (TLRs). Whether this mechanism, thought to require the participation of topoisomerases and/or DNAses and thus far only described in epididymal mouse sperm, can be triggered in ejaculated sperm is yet to be elucidated. The current study aimed to determine if exposure of pig ejaculated sperm to divalent ions (Mn^2+^ and Mg^2+^) activates SCF, and whether this has any impact on sperm function and survival. For this purpose, sperm DNA integrity was evaluated through the Comet assay and Pulsed Field Gel Electrophoresis (PFGE); sperm motility and agglutination were assessed with computer assisted sperm analysis (CASA); and sperm viability and levels of total reactive oxygen species (ROS) and superoxides were determined through flow cytometry.

**Results:**

Incubation with Mn^2+^/Ca^2+^ activated SCF in a dose-dependent (*P* < 0.05) albeit not time-dependent manner (*P* > 0.05); in contrast, Mg^2+^/Ca^2+^ only triggered SCF at high concentrations (50 mM). The PFGE revealed that, when activated by Mn^2+^/Ca^2+^ or Mg^2+^/Ca^2+^, SCF generated DNA fragments of 33–194 Kb, compatible with the size of one or multiple toroids. Besides, Mn^2+^/Ca^2+^ affected sperm motility in a dose-dependent manner (*P* < 0.05), whereas Mg^2+^/Ca^2+^ only impaired this variable at high concentrations (*P* < 0.05). While this effect on motility was concomitant with an increase of agglutination, neither viability nor ROS levels were affected by Mn^2+^/Ca^2+^ or Mg^2+^/Ca^2+^ treatments.

**Conclusion:**

Mn^2+^/Ca^2+^ and Mn^2+^/Ca^2+^ were observed to induce SCF in ejaculated sperm, resulting in DNA cleavage at TLRs. The activation of this mechanism by an intracellular, non-oxidative factor sheds light on the events taking place during sperm cell death.

## Background

Sperm DNA breaks are one of the most important chromatin alterations that underlie male infertility and contribute to pregnancy loss [1–4]. Since its inception, the study of sperm DNA fragmentation has focused on elucidating which mechanisms cause DNA breaks, as not only does this help understand how they may impact reproductive outcomes [5–8], but also addresses whether they may be prevented both in vivo and in vitro [9, 10].

Sperm cells reach a high degree of differentiation after spermiogenesis, a process during which chromatin undergoes extreme remodeling, replacing most of the histones by protamines [11–13]. This specific packaging of nuclear material leads to the formation of toroidal structures lengthening about 50 Kb [14], which are stabilized by intra- and inter-protamine interactions [15, 16]. The packaging of sperm DNA into toroids containing protamines provides a very high degree of condensation, thus protecting the paternal genome from genotoxic damage [17, 18]. According to the most accepted model for chromatin organization, toroids are linked with each other and to the nuclear matrix through Toroid Linker Regions (TLR), which are less condensed and contain histones instead of protamines [19–21]. A high incidence of double-stranded DNA breaks (DSB) in TLRs is associated to an increased risk of recurrent pregnancy loss and implantation failure [22], suggesting a vital function for the genes located in these regions during early embryo development. Furthermore, specific TLRs attached to the nuclear scaffold have been suggested to play a crucial role in the initiation of paternal DNA replication [23]. Moreover, previous research demonstrated that, in addition to the fact that TLRs are nuclease-sensitive [17, 24, 25], in vitro incubation of epididymal and vas deferens sperm with their own fluid supplemented with Mn^2+^/Ca^2+^ induces a mechanism known as Sperm Chromatin Fragmentation (SCF), which causes double-stranded DNA breaks in TLRs [20, 26]. Noticeably, the DSBs induced by the in vitro activation of SCF remain attached to the nuclear matrix, which suggests that such a binding could be part of a mechanism of the zygote to repair its DNA [25] and reinforces the importance of the biological function of TLRs. Regarding the molecular mechanism originating SCF in vitro, although it has been suggested to result from endogenous nuclease activation [19, 20], the possibility that a topoisomerase associated to the nuclear matrix causes these DSBs should not be excluded [25, 27, 28].

A recent study conducted in vasectomized mice demonstrated, for the first time, that SCF also exists as an in vivo mechanism contributing to natural sperm degradation after certain time of storage in the epididymis [29]. This mechanism would inactivate sperm chromatin and prevent defective sperm that have been stored for a long period in the epididymis from being functional. While that study supported that SCF can be activated in epididymal and vas deferens mouse sperm, whether ejaculated sperm also have the ability to trigger this process is yet to be investigated. Remarkably, this would provide a broader picture about how DNA damage occurs in mature sperm as, whereas most DNA breaks are thought to result from oxidative stress [30, 31], those induced by SCF would certainly be produced by a separate mechanism. In the light of the aforementioned, the present work aimed to: (a) determine if the SCF mechanism is also active in ejaculated sperm, using the pig as an animal model; (b) interrogate if Mg^2+^ ions can elicit the mechanism, in a similar fashion to Mn^2+^; and (c) address if the activation of this DNA-fragmentation mechanism entails other consequences for sperm function and survival.

## Results

### Sperm DNA breaks can be induced in ejaculated sperm through intracellular divalent ions

The first experiment aimed to assess if DNA breaks can be induced in ejaculated sperm in vitro and, if so, if this relies upon intracellular or extracellular factors. For this purpose, permeabilized and non-permeabilized samples were incubated with different concentrations of Mn^2+^/Ca^2+^ or Mg^2+^/Ca^2+^ for different incubation times.

**Fig. 1:**
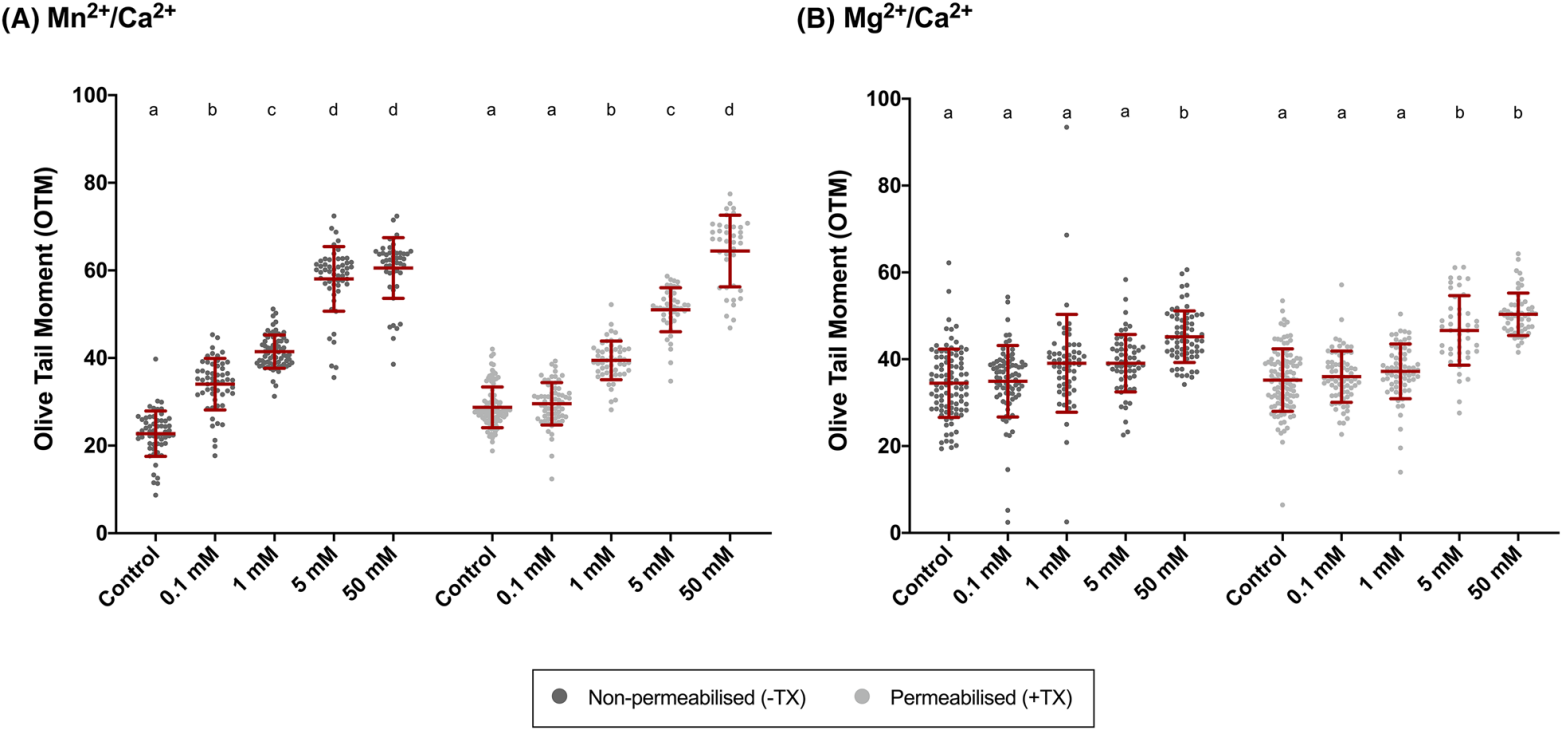
Comet assay Olive Tail Moment (OTM) after incubation of sperm with different concentrations (0 mM, 0.1 mM, 1 mM, 5 mM and 50 mM) of Mn^2+^/Ca^2+^ (**A**) or Mg^2+^/Ca^2+^ (**B**) in non-permeabilized (−TX) and permeabilized (+ TX) samples. Different letters indicate significant differences (*P* ≤ 0.05) between concentrations of Mn^2+^/Ca^2+^ (A) or Mg^2+^/Ca^2+^ (**B**) within non-permeabilized (−TX) and permeabilized (+TX) samples. Comparisons between non-permeabilized (−TX) and permeabilized (+TX) samples, or between ions (Mn^2+^/Ca^2+^ vs. Mg^2+^/Ca^2+^) are not depicted

**Fig. 2:**
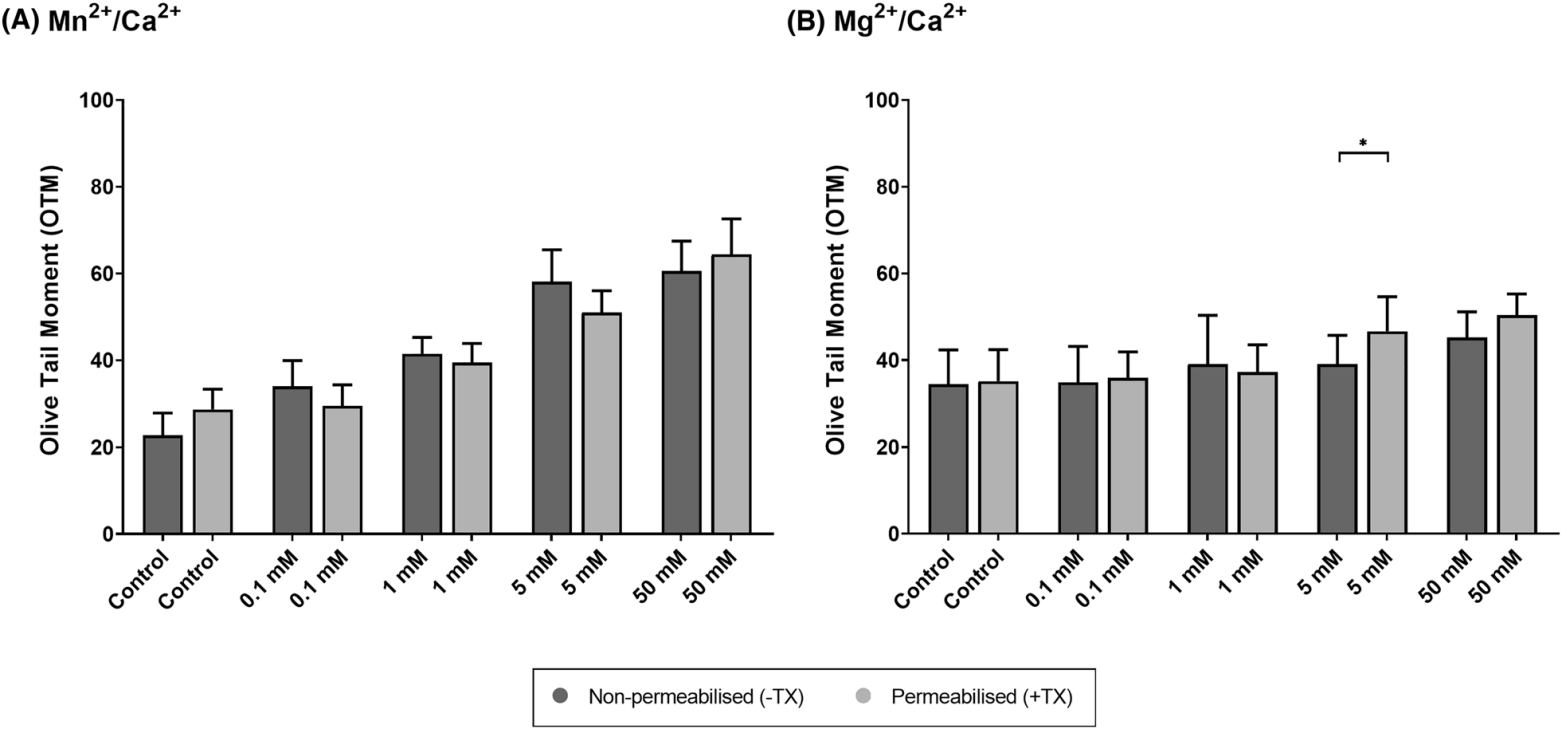
Comet assay Olive Tail Moment (OTM) after incubation of sperm with different concentrations of Mn^2+^/Ca^2+^ (**A**) or Mg^2+^/Ca^2+^ (**B**) (0 mM, 0.1 mM, 1 mM, 5 mM and 50 mM), in non-permeabilized (−TX) and permeabilized (+TX) samples. (*) indicates significant differences between non-permeabilized (−TX) and permeabilized (+TX) samples (*P* ≤ 0.05)

Dose–response results for Mn^2+^/Ca^2+^ and Mg^2+^/Ca^2+^ treatments are shown in Fig. 1 and Table 1. On the one hand, Mn^2+^/Ca^2+^ exerted a dose-dependent effect on the generation of DNA breaks, in both permeabilized and non-permeabilized samples. In the latter, there were significant differences between Mn^2+^/Ca^2+^ treatments (*P* < 0.05), except between the two highest concentrations (Fig. 1A). In contrast, incubation with Mg^2+^/Ca^2+^ showed no dose-dependent effect, and an increased incidence of DNA breaks was only detected when comparing incubations with the highest Mg^2+^/Ca^2+^ concentrations (Fig. 1B). Specifically, statistical differences were only observed between the control and 5 mM Mg^2+^/Ca^2+^ (*P* = 0.010), and between 50 mM Mg^2+^/Ca^2+^ and all the other treatments (*P* < 0.05), in both non-permeabilized (−TX) and permeabilized samples (+TX) (*P* < 0.05). As no significant differences between permeabilized and non-permeabilized samples were found after incubation with Mn^2+^/Ca^2+^ or Mg^2+^/Ca^2+^ (*P* > 0.05) (Fig. 2), it seemed that an intracellular component triggered the SCF mechanism and thus generated DNA breaks.

**Table 1:** Olive Tail Moment (OTM) after incubation with different concentrations of Mn^2+^/Ca^2+^ or Mg^2+^/Ca^2+^ (0 mM, 0.1 mM, 1 mM, 5 mM and 50 mM) in non-permeabilized (−TX) and permeabilized (+TX) samples

	DNA damage (olive tail moment)	
	Non-permeabilized (−TX)	Permeabilized (+TX)
(A) Mn^2+^/Ca^2+^		
Control (0 mM)	22.72 ± 5.18^a^	28.73 ± 4.65^a^
0.1 mM	34.03 ± 5.88^b^	29.55 ± 4.84^a^
1 mM	41.45 ± 3.81^c^	39.45 ± 4.41^b^
5 mM	58.07 ± 7.38^d^	51.01 ± 5.02^c^
50 mM	60.53 ± 6.93^d^	64.41 ± 8.17^d^
(B) Mg^2+^/Ca^2+^		
Control (0 mM)	34.48 ± 7.86^a^	35.20 ± 7.20^a^
0.1 mM	34.93 ± 8.22^a^	35.99 ± 5.90^a^
1 mM	39.06 ± 11.26^a^	37.21 ± 6.32^a^
5 mM	39.08 ± 6.61^a^	46.63 ± 8.01^b^
50 mM	45.18 ± 5.94^c^	50.35 ± 4.89^b^

Different letters indicate significant differences (*P* ≤ 0.05) between concentrations of Mn^2+^/Ca^2+^ (A) or Mg^2+^/Ca^2+^ (B) within non-permeabilized (−TX) and permeabilized (+TX) samples. Comparisons between non-permeabilized (−TX) and permeabilized (+TX) samples, or between ions (Mn^2+^/Ca^2+^ vs. Mg^2+^/Ca^2+^) are not depicted

**Fig. 3:**
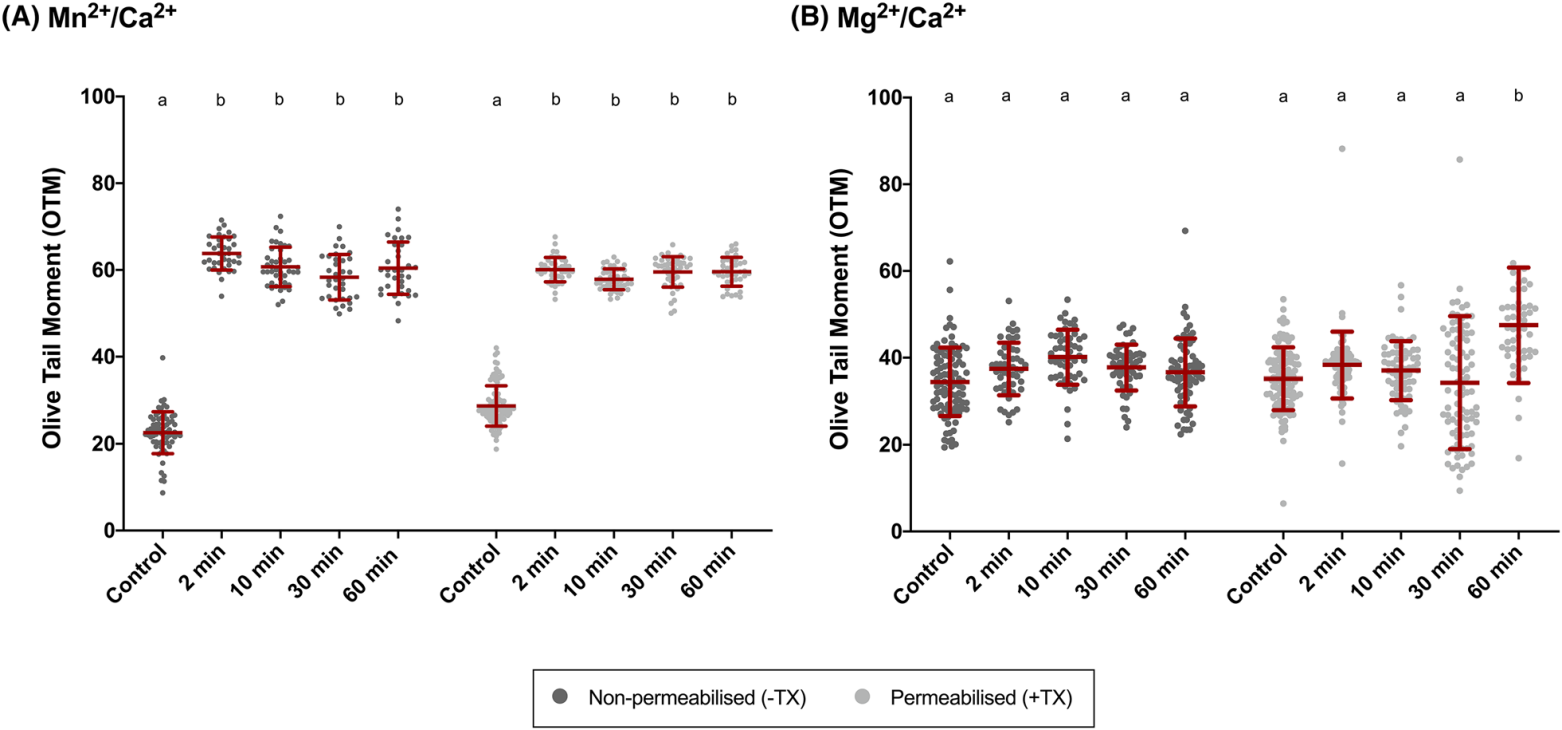
Comet assay Olive Tail Moment (OTM) after incubation of sperm with 10 mM of Mn^2+^/Ca^2+^ (**A**) or 10 mM Mg^2+^/Ca^2+^ (**B**) for different incubation times (0 min, 2 min, 10 min, 30 min, 60 min) in non-permeabilized (−TX) and permeabilized (+TX) samples. Different letters indicate significant differences (*P* ≤ 0.05) between incubation times within non-permeabilized (−TX) and permeabilized (+TX) samples. Comparisons between non-permeabilized (−TX) and permeabilized (+TX) samples, or between ions (Mn^2+^/Ca^2+^ vs. Mg^2+^/Ca^2+^) are not depicted

**Fig. 4:**
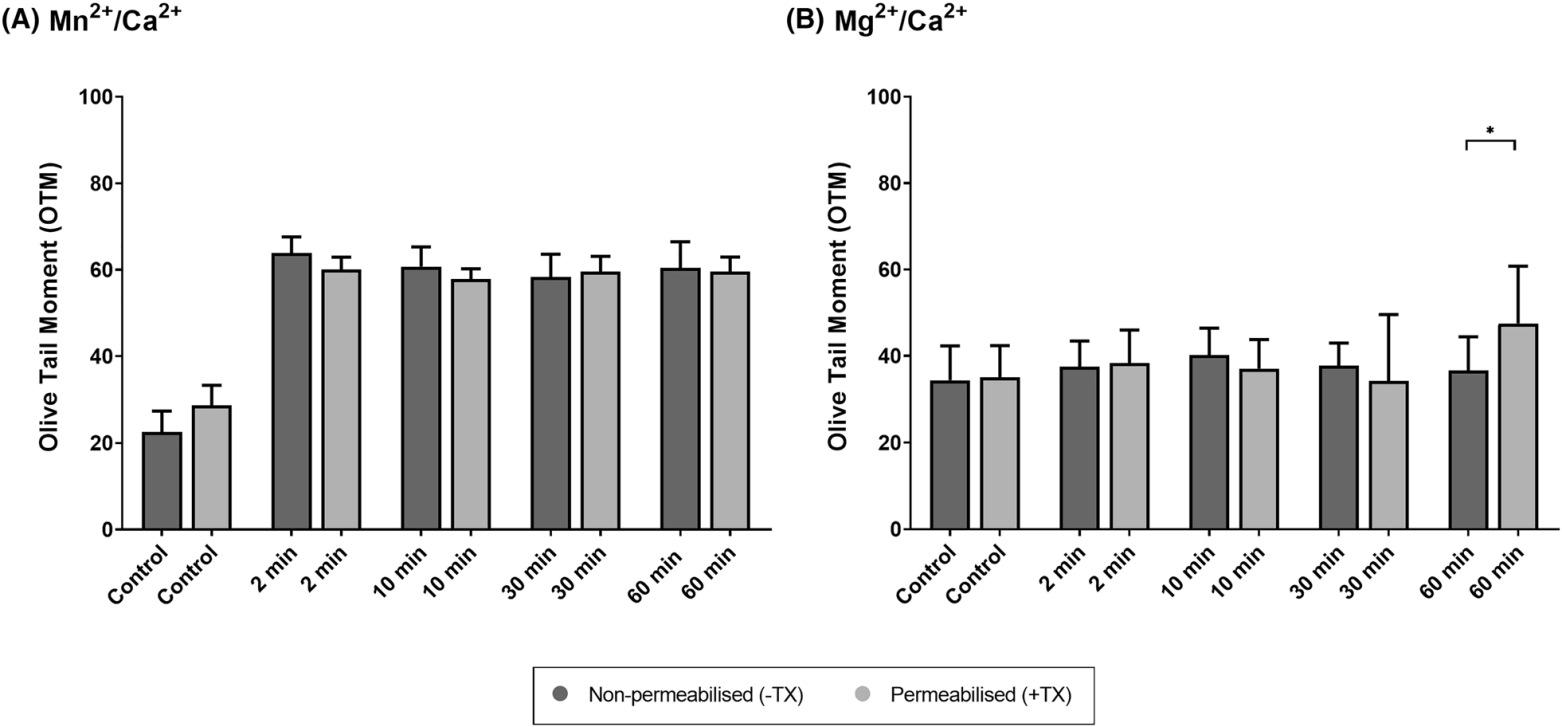
Comet assay Olive Tail Moment (OTM) after incubation of sperm with 10 mM of Mn^2+^/Ca^2+^ (**A**) or 10 mM Mg^2+^/Ca^2+^ (**B**) for different incubation times (0 min, 2 min, 10 min, 30 min, 60 min), in non-permeabilized (−TX) and permeabilized (+TX) samples. (*) indicates significant differences between non-permeabilized (−TX) and permeabilized (+TX) samples (*P* ≤ 0.05)

On the other hand, and as shown in Fig. 3 and Table 2, there was no effect of incubation time on the activation of the SCF mechanism, regardless of whether samples were permeabilized or non-permeabilized. In effect, incubation with 5 mM Mn^2+^/Ca^2+^ for different times (2 min, 10 min, 30 min and 60 min) led to similar DNA damage (*P* > 0.05), which was greater than in the control (*P* > 0.05). In the case of Mg^2+^, only the longest incubation (60 min) with Mg^2+^/Ca^2+^ appeared to evoke SCF, as solely this period was significantly different from the others (2 min, 10 min and 30 min) and from the control (*P* < 0.05). In short, while the effect of incubation time did not differ between permeabilized and non-permeabilized sperm in Mn^2+^/Ca^2+^ treatments, the extent of DNA damage was greater in permeabilized than in non-permeabilized samples after incubation with Mg^2+^/Ca^2+^ for 60 min (*P* < 0.05) (Fig. 4).

**Table 2:** Olive Tail Moment (OTM) after incubation with 10 mM Mn^2+^/Ca^2+^ (A) or 10 mM Mg^2+^Ca^2+^ (B) for different incubation times (0 min, 2 min, 10 min, 30 min, 60 min), in non-permeabilized (−TX) and permeabilized (+TX) samples

	DNA damage (olive tail moment)	
	Non-permeabilized (−TX)	Permeabilized (+TX)
(A) 10 mM Mn^2+^/Ca^2+^		
Control (0 min)	22.57 ± 4.82^a^	28.73 ± 4.65^a^
2 min	63.80 ± 3.80^b^	60.07 ± 2.82^b^
10 min	60.71 ± 4.55^b^	57.85 ± 2.39^b^
30 min	58.34 ± 5.24^b^	59.55 ± 3.52^b^
60 min	60.42 ± 6.03^b^	59.59 ± 3.33^b^
(B) 10 mM Mg^2+^/Ca^2+^		
Control (0 min)	34.48 ± 7.86^a^	35.20 ± 7.20^a^
2 min	37.44 ± 6.01^a^	38.35 ± 7.67^a^
10 min	40.16 ± 6.29^a^	37.04 ± 6.75^a^
30 min	37.76 ± 5.24^a^	34.30 ± 15.30^a^
60 min	36.63 ± 7.79^a^	47.51 ± 13.27^b^

Different letters indicate significant differences (*P* ≤ 0.05) between incubation times within non-permeabilized (−TX) and permeabilized (+TX) samples. Comparisons between non-permeabilized (−TX) and permeabilized (+TX) samples, or between ions (Mn^2+^/Ca^2+^ vs. Mg^2+^/Ca^2+^) are not depicted

### Incubation with Mn^2+^/Ca^2+^ or Mg^2+^/Ca^2+^ triggers the SCF mechanism in ejaculated sperm in vitro

**Fig. 5:**
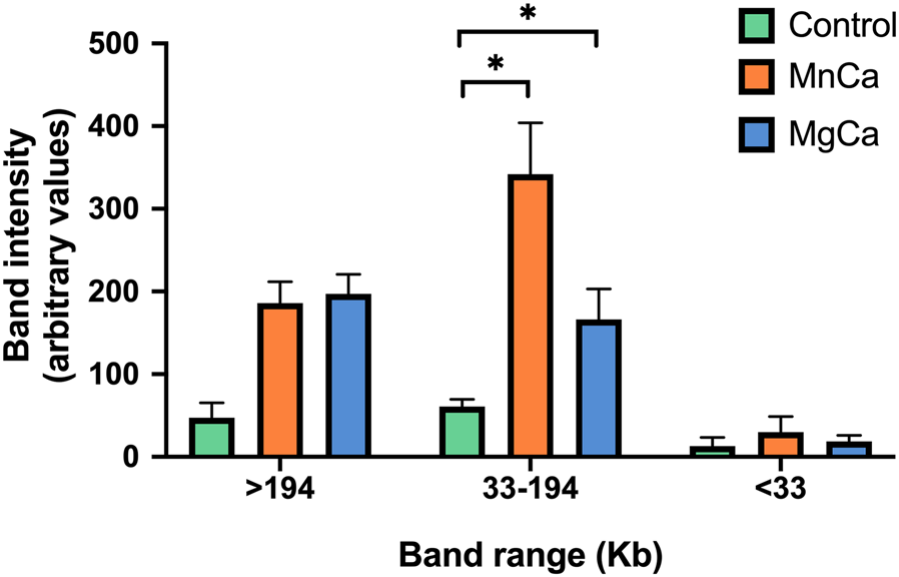
Analysis of Pulsed Field Gel Electrophoresis DNA (PGFE) intensity. Three types of DNA fragments differing in size were identified: (i) DNA fragments > 194 Kb in length, compatible to mostly intact DNA (i.e., where SCF did not occur); (ii) DNA fragments ranging between 33 and 194 Kb in length, compatible to DNA packed into one to four toroids; and (iii) DNA fragments > 33 kb, compatible to sizes smaller than one toroid. (*) indicates significant differences between the control and treatments (*P* ≤ 0.05)

After confirming that incubation with Mn^2+^/Ca^2+^ and Mg^2+^/Ca^2+^ induces DNA breaks, pulsed-field gel electrophoresis was run to determine the sizes of the resulting fragments. Noticeably, incubation with Mn^2+^/Ca^2+^ and Mg^2+^/Ca^2+^ increased (*P* < 0.05) the number of DNA fragments ranging between 33 and 194 Kb, a size that would be compatible with the DNA condensed into one to four toroids (Fig. 5). This would suggest that Mn^2+^/Ca^2+^ and Mg^2+^/Ca^2+^ trigger SCF in ejaculated pig sperm in vitro*,* in a similar fashion to that observed before in epididymal and vas deferens mouse sperm in vivo [29].

### Mn^2+^/Ca^2+^ impairs sperm motility in a dose-dependent manner, whereas Mg^2+^/Ca^2+^ only has an effect at high concentrations

**Fig. 6:**
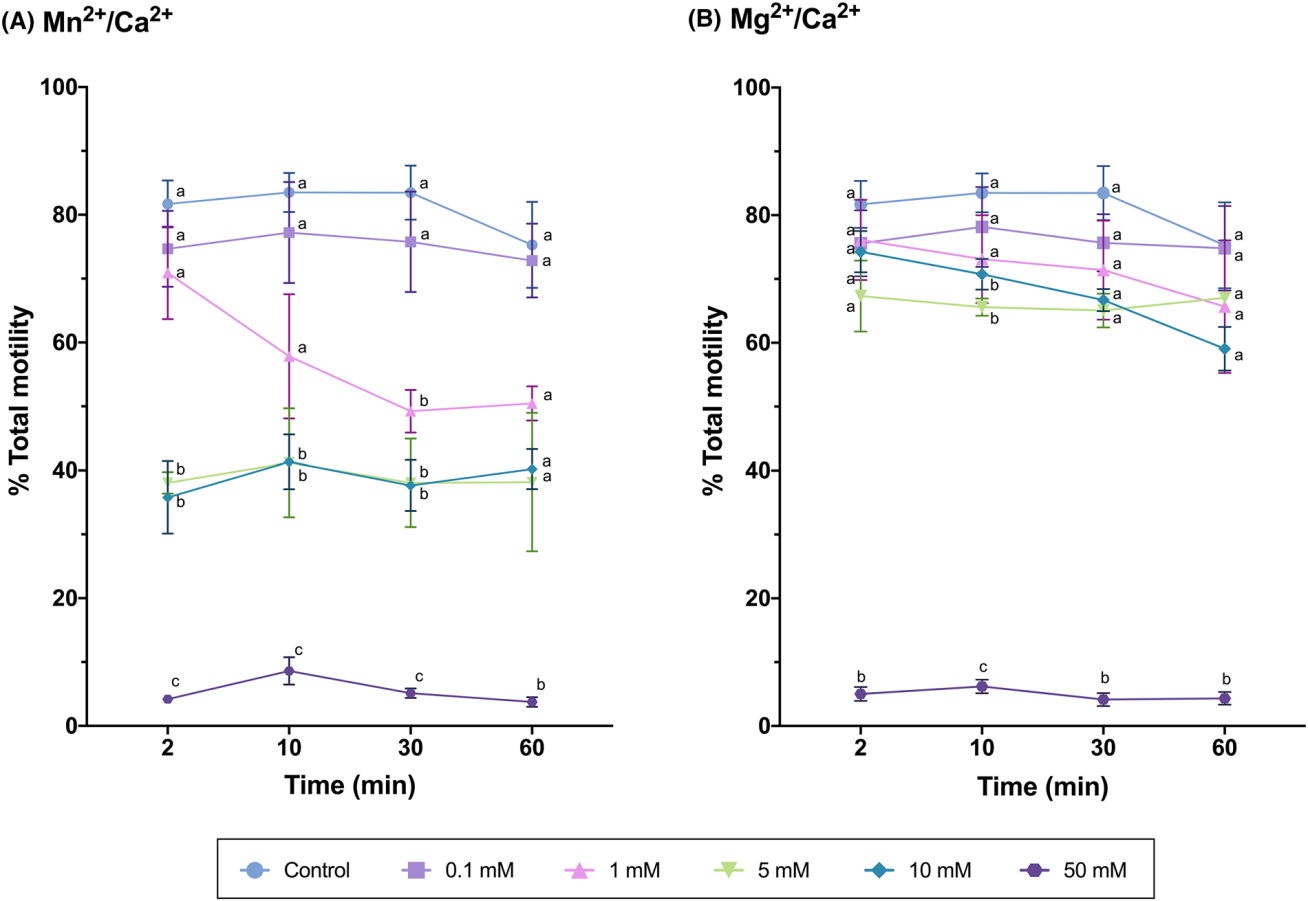
Percentages of motile sperm after incubation with Mn^2+^/Ca^2+^ (**A**) or Mg^2+^/Ca^2+^ (**B**) at different concentrations (0 mM, 0.1 mM, 1 mM, 5 mM, 10 mM and 50 mM) and for different incubation times (0 min, 2 min, 10 min, 30 min, 60 min). Different letters indicate significant differences (*P* ≤ 0.05) between concentrations of Mn^2+^/Ca^2+^ (**A**) or Mg^2+^/Ca^2+^ (**B**) at a given time point. Differences between time points within a given concentration are not shown

In addition to sperm DNA integrity, the effects of Mn^2+^/Ca^2+^ and Mg^2+^/Ca^2+^ on sperm motility, agglutination, viability and levels of total ROS and superoxides were also examined. In the case of sperm motility, and as shown in Fig. 6, Mn^2+^/Ca^2+^ caused a concentration-dependent reduction of sperm motility, which was very obvious at high concentrations; in contrast, no effect of incubation time was observed (Fig. 6A; Table 3). Incubation with Mg^2+^/Ca^2+^ also induced a drastic drop of sperm motility, but only at the highest concentration (50 mM) (*P* < 0.05), as the other concentrations (0.1 mM, 1 mM, 5 mM and 10 mM) did not differ from the control (Fig. 6B; Table 4) (*P* > 0.05).

**Table 3:** Percentages of motile sperm, viable sperm, sperm with high intracellular ROS, sperm with high intracellular superoxide levels and sperm agglutination after incubation with different concentrations of Mn^2+^/Ca^2+^ (0 mM, 0.1 mM, 1 mM, 5 mM, 10 mM and 50 mM) and for different incubation times (0 min, 2 min, 10 min, 30 min, 60 min)

	Motility (%motile sperm)	Viability (%viable sperm)	ROS (%sperm with high intracellular ROS)	Superoxides (%sperm with high intracellular superoxides)	Agglutination (%agglutinated sperm)
2 min					
Control (0 mM)	81.69 ± 6.36^a^	50.93 ± 12.74	8.01 ± 6.28	36.95 ± 13.67	7.48 ± 2.05^a^
0.1 mM	74.66 ± 10.29^a^	54.13 ± 11.77	9.80 ± 7.86	36.28 ± 14.19	15.97 ± 6.69^a^
1 mM	70.91 ± 12.54^a^	55.96 ± 8.27	11.35 ± 8.07	39.13 ± 11.86	38.70 ± 2.74^b^
5 mM	38.06 ± 2.91^b^	53.41 ± 8.02	12.03 ± 5.48	35.00 ± 11.59	73.42 ± 2.54^c^
10 mM	35.78 ± 9.80^b^	49.42 ± 9.00	11.88 ± 7.28	39.45 ± 11.88	88.88 ± 2.97^c^
50 mM	4.19 ± 0.61^c^	34.21 ± 22.00	3.95 ± 1.99	24.90 ± 9.42	99.34 ± 0.84^d^
10 min					
Control (0 mM)	83.49 ± 5.29^a^	54.70 ± 16.13	8.38 ± 3.84	46.28 ± 19.39	8.81 ± 1.44^a^
0.1 mM	77.22 ± 13.69^a^	58.15 ± 15.55	12.12 ± 4.09	47.35 ± 21.23	17.67 ± 5.73^a^
1 mM	57.84 ± 16.84^a^	58.77 ± 14.60	14.61 ± 5.59	46.70 ± 16.20	41.52 ± 4.67^b^
5 mM	41.19 ± 14.74^b^	52.37 ± 3.30	11.90 ± 2.14	44.87 ± 13.15	67.43 ± 5.41^b^
10 mM	41.33 ± 7.41^b^	53.74 ± 7.88	8.12 ± 1.99	42.28 ± 9.42	86.14 ± 5.37^c^
50 mM	8.61 ± 3.72^c^	39.89 ± 19.33	5.25 ± 6.15	34.59 ± 8.42	96.99 ± 1.95^c^
30 min					
Control (0 mM)	83.47 ± 7.34^a^	60.98 ± 11.23	6.46 ± 2.40	34.65 ± 15.04	11.02 ± 1.43^a^
0.1 mM	75.76 ± 13.61^a^	62.79 ± 6.32	11.05 ± 5.29	32.91 ± 12.34	19.26 ± 7.07^a^
1 mM	49.24 ± 5.77^b^	61.28 ± 8.99	11.45 ± 4.58	37.68 ± 14.78	40.28 ± 6.25^a^
5 mM	38.05 ± 11.98^b^	60.43 ± 8.92	11.15 ± 4.97	33.77 ± 7.83	74.32 ± 2.76^b^
10 mM	37.65 ± 6.89^b^	48.34 ± 23.48	5.69 ± 4.98	35.01 ± 10.71	84.95 ± 3.83^b^
50 mM	5.12 ± 1.29^c^	22.36 ± 27.83	4.32 ± 4.43	36.42 ± 18.03	93.63 ± 8.18^b^
60 min					
Control (0 mM)	75.29 ± 11.65^a^	49.09 ± 17.94	4.91 ± 4.41	45.43 ± 11.86	19.00 ± 12.95^a^
0.1 mM	72.83 ± 10.00^a^	44.80 ± 18.50	4.68 ± 5.28	48.65 ± 4.46	25.43 ± 5.34^a^
1 mM	50.46 ± 4.64^a^	50.51 ± 10.83	5.01 ± 6.06	53.38 ± 9.25	37.47 ± 4.27^a^
5 mM	38.17 ± 18.72^a^	42.61 ± 19.33	5.37 ± 6.96	57.51 ± 20.88	71.71 ± 2.98^b^
10 mM	40.20 ± 5.43^a^	47.41 ± 6.94	5.82 ± 5.40	53.82 ± 7.85	83.80 ± 4.90^b^
50 mM	3.75 ± 1.30^b^	17.54 ± 28.07	2.44 ± 3.68	46.96 ± 28.95	96.87 ± 3.02^c^

Different letters indicate significant differences (*P* ≤ 0.05) between concentrations of Mn^2+^/Ca^2+^ at a given time point. Values without letters indicate no statistical differences between treatments within a given time point

**Table 4:** Percentages of motile sperm, viable sperm, sperm with high intracellular ROS, sperm with high intracellular superoxide levels and sperm agglutination after incubation with different concentrations of Mg^2+^/Ca^2+^ (0 mM, 0.1 mM, 1 mM, 5 mM, 10 mM and 50 mM) and for different incubation times (0 min, 2 min, 10 min, 30 min, 60 min)

	Motility (%motile sperm)	Viability (%viable sperm)	ROS (%sperm with high intracellular ROS)	Superoxides (%sperm with high intracellular superoxides)	Agglutination (%agglutinated sperm)
**2 min**					
Control (0 mM)	81.69 ± 6.36^a^	50.93 ± 12.74	8.01 ± 6.28	36.95 ± 13.67	7.48 ± 2.05^a^
0.1 mM	75.59 ± 8.97^a^	53.82 ± 13.40	8.30 ± 5.73	32.18 ± 14.13	10.85 ± 3.35^a^
1 mM	76.12 ± 10.89^a^	56.87 ± 12.12	7.01 ± 6.31	32.42 ± 9.04	17.18 ± 5.10^a^
5 mM	67.32 ± 9.64^a^	55.09 ± 6.24	11.50 ± 7.77	40.16 ± 19.29	36.70 ± 19.72^a^
10 mM	74.28 ± 5.60^a^	44.76 ± 8.49	14.25 ± 11.05	56.46 ± 13.32	59.09 ± 6.82^b^
50 mM	5.02 ± 1.89^b^	38.31 ± 7.94	12.61 ± 7.60	46.77 ± 8.64	84.77 ± 5.56^b^
10 min					
Control (0 mM)	83.49 ± 5.29^a^	54.70 ± 16.13	8.38 ± 3.84	46.28 ± 19.39	8.81 ± 1.44^a^
0.1 mM	78.16 ± 10.8^a^	58.95 ± 16.71	11.90 ± 5.12	38.04 ± 13.45	10.41 ± 4.47^a^
1 mM	73.11 ± 11.92^a^	60.16 ± 14.58	12.23 ± 5.23	49.00 ± 18.74	15.11 ± 2.44^a^
5 mM	65.59 ± 2.35^b^	47.63 ± 10.77	13.56 ± 7.69	61.78 ± 5.44	34.24 ± 7.84^a^
10 mM	70.74 ± 4.18^b^	48.81 ± 16.93	12.80 ± 4.90	64.50 ± 9.59	57.82 ± 4.85^b^
50 mM	6.19 ± 1.84^c^	47.90 ± 6.50	6.34 ± 3.22	49.68 ± 6.26	86.15 ± 3.89^b^
30 min					
Control (0 mM)	83.47 ± 7.34^a^	60.98 ± 11.23	6.46 ± 2.40	34.65 ± 15.04	11.02 ± 1.43^a^
0.1 mM	75.67 ± 7.75^a^	63.44 ± 8.67	9.87 ± 2.33	32.02 ± 14.21	14.80 ± 3.63^a^
1 mM	71.39 ± 13.43^a^	58.29 ± 10.18	9.85 ± 2.68	36.89 ± 15.05	20.17 ± 5.94^a^
5 mM	65.06 ± 4.57^a^	55.97 ± 9.46	12.26 ± 4.55	43.39 ± 16.35	34.56 ± 5.70^b^
10 mM	66.69 ± 2.99^a^	42.57 ± 26.12	12.07 ± 3.73	45.16 ± 18.95	61.23 ± 5.97^b^
50 mM	4.14 ± 1.74^b^	38.20 ± 9.45	6.04 ± 2.72	39.00 ± 10.36	81.44 ± 2.36^c^
60 min					
Control (0 mM)	75.29 ± 11.65^a^	49.09 ± 17.94	4.91 ± 4.41	45.43 ± 11.86	19.00 ± 12.95^a^
0.1 mM	74.82 ± 11.46^a^	42.00 ± 20.10	5.00 ± 5.68	49.88 ± 9.71	19.35 ± 5.11^a^
1 mM	65.67 ± 17.99^a^	50.38 ± 11.28	5.31 ± 5.31	53.14 ± 6.49	24.11 ± 6.06^a^
5 mM	67.04 ± 0.97^a^	44.39 ± 16.67	5.64 ± 5.67	63.49 ± 8.07	33.15 ± 6.93^a^
10 mM	59.06 ± 5.92^a^	31.52 ± 15.86	4.68 ± 3.55	65.53 ± 24.71	56.22 ± 11.57^b^
50 mM	4.32 ± 1.71^b^	33.24 ± 5.47	3.12 ± 2.97	56.88 ± 16.61	77.70 ± 7.48^c^

Different letters indicate significant differences (*P* ≤ 0.05) between concentrations of Mg^2+^/Ca^2+^ at a given time point. Values without letters indicate no statistical differences between treatments within a given time point

### Incubation with Mn^2+^/Ca^2+^ or Mg^2+^/Ca^2+^ has no effect on sperm viability

**Fig. 7:**
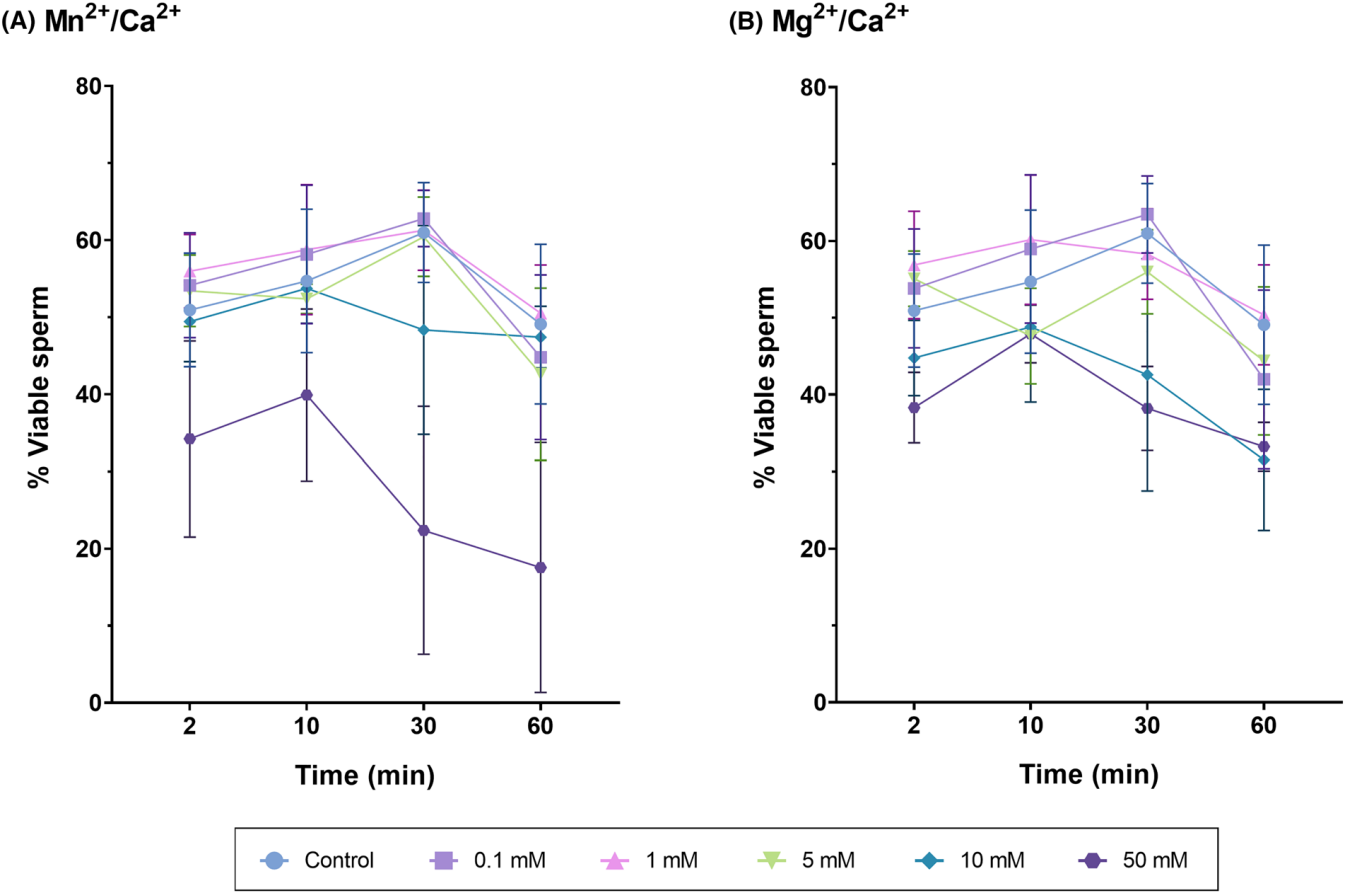
Percentages of viable sperm after incubation with Mn^2+^/Ca^2+^ (**A**) or Mg^2+^/Ca^2+^ (**B**) at different concentrations (0 mM, 0.1 mM, 1 mM, 5 mM, 10 mM and 50 mM) and for different incubation times (0 min, 2 min, 10 min, 30 min, 60 min). No significant differences between treatments at any time point were observed

Neither Mn^2+^/Ca^2+^ nor Mg^2+^/Ca^2+^ were found to have a significant effect on sperm viability (*P* > 0.05; Fig. 7; Tables 3, 4). Yet, there was a trend for higher concentrations (Mn^2+^/Ca^2+^ 50 mM and Mg^2+^/Ca^2+^ 10 and 50 mM) and longer incubation times (60 min) to reduce the proportion of viable sperm.

### SCF is not related to oxidative stress, as neither total ROS nor superoxide levels are altered following incubation of sperm with Mn^2+^/Ca^2+^ or Mg^2+^/Ca^2+^

**Fig. 8:**
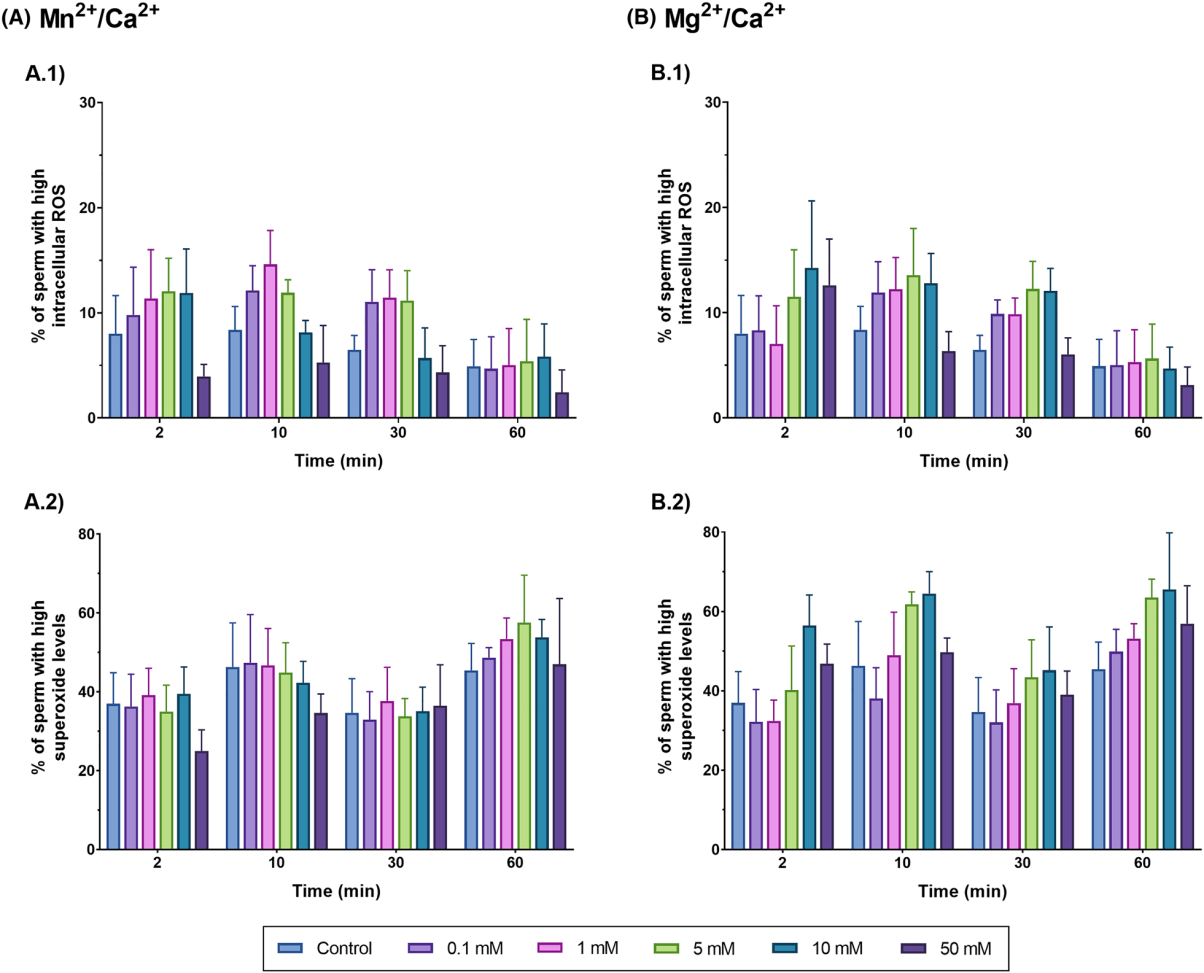
Total ROS and superoxide levels after incubation of sperm with Mg^2+^/Ca^2+^ (**A.1** and **A.2**) or Mn^2+^/Ca^2+^ (**B.1** and **B.2**) at different concentrations (0 mM, 0.1 mM, 1 mM, 5 mM, 10 mM and 50 mM) and for different incubation times (0 min, 2 min, 10 min, 30 min, 60 min). Differences between time points within a given concentration are not shown

Incubation of sperm with Mn^2+^/Ca^2+^ or Mg^2+^/Ca^2+^ did not increase intracellular levels of total ROS or superoxides in sperm (Fig. 8; Tables 3, 4) (*P* > 0.05). The fact that ROS were not increased upon activation of SCF suggests that this mechanism does not rely upon oxidative stress.

### Incubation with both Mn^2+^/Ca^2+^ and Mg^2+^/Ca^2+^ induces sperm agglutination

**Fig. 9:**
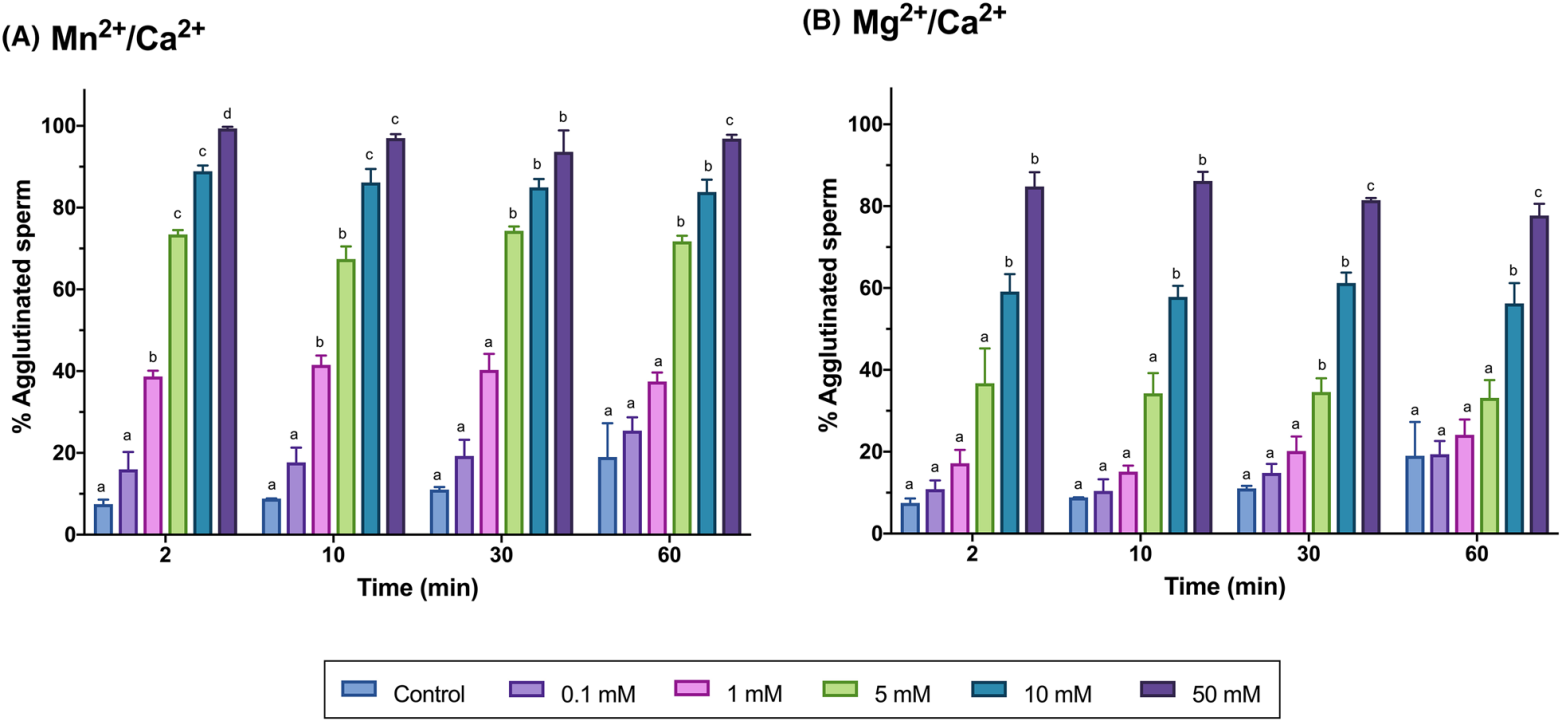
Percentages of agglutinated sperm after incubation with Mn^2+^/Ca^2+^ (**A**) or Mg^2+^/Ca^2+^ (**B**) at different concentrations (0 mM, 0.1 mM, 1 mM, 5 mM, 10 mM and 50 mM) and for different incubation times (0 min, 2 min, 10 min, 30 min, 60 min). Different letters indicate significant differences (*P* ≤ 0.05) between concentrations of Mn^2+^/Ca^2+^ (**A**) or Mg^2+^/Ca^2+^ (**B**) at a given time point. Differences between time points within a given concentration are not shown

As shown in Fig. 9, and Tables 3, 4, the proportion of agglutinated sperm increased in a dose-dependent manner following incubation with Mn^2+^/Ca^2+^ or Mg^2+^/Ca^2+^ (*P* < 0.05). Conversely, there was no influence from the incubation time (*P* > 0.05). Remarkably, a massive sperm agglutination was observed at high concentrations of Mn^2+^/Ca^2+^ (10 mM and 50 mM), with the proportion of agglutination being greater than 80%.

## Discussion

The SCF mechanism is an intrinsic process through which sperm degrade their own DNA, producing toroid-sized fragments [19, 20]. This mechanism was first described in epididymal and vas deferens mouse sperm, where not only was demonstrated to be triggered in vitro after incubation with Mn^2+^/Ca^2+^ [20, 26, 28], but also in vivo, as a way to prevent the functionality of sperm chromatin after vasectomy [29]. In mouse, SCF was found to be mediated via the activation of a nuclease present in the epididymis and vas deferens sperm and a topoisomerase IIB [27, 28], after incubation with epididymal or vas deferens fluids supplemented with Mn^2+^. It remained, notwithstanding, unknown if SCF can be triggered in vitro in ejaculated sperm and, if so, whether the mechanism identified in mouse differs from that occurring in other mammalian species, which is why the pig was chosen as a model herein.

The present study demonstrates, for the first time, that the SCF mechanism can also be elicited in ejaculated sperm in vitro after incubation with Mn^2+^/Ca^2+^, with the size of fragments generated by the breaks being compatible to those of the DNA condensed into toroidal structures. While this supports that this process is similar to that previously reported in epididymal and vas deferens mouse sperm, it is worth noting that, in the present work, SCF could be activated even in non-permeabilized sperm. This would suggest that, at least in ejaculated sperm, a transduction pathway including surface receptors and an intracellular machinery might be involved in this mechanism. In addition, while both Mn^2+^/Ca^2+^ and Mg^2+^/Ca^2+^ were able to elicit SCF, the concentration required to do so was lower in the former than in the latter. This suggests that, although the pathway triggered is not ion-specific, the affinity of the enzymes involved for Mn^2+^ and Mg^2+^ is different. Finally, in vitro activation of SCF in ejaculated sperm, despite not altering sperm viability or ROS levels, caused a reduction of sperm motility and increased agglutination. All these findings are separately discussed below.

First, and regarding DNA fragmentation, results obtained for ejaculated sperm in this study were in agreement with those previously reported in epididymal and vas deferens mouse sperm [25, 27–29, 32]. In that earlier research, Mn^2+^ at a concentration of 10 mM, with or without Ca^2+^, was found to trigger the SCF mechanism. Herein, it was observed that even a low concentration of Mn^2+^/Ca^2+^ was enough to activate SCF without the presence of a specific surrounding fluid; yet, the effect of this cation was dose-dependent, so that the greater the concentration of Mn^2+^/Ca^2+^ the higher the incidence of DNA breaks. In addition, the effect of Mn^2+^/Ca^2+^ was fast and very apparent as early as 2 min post-incubation, and a longer incubation period did not result in a higher incidence of sperm DNA breaks. Based on this, one can suggest that the activation of the SCF mechanism, at least in vitro, relies on the concentration of Mn^2+^/Ca^2+^ but not on the incubation period. Because such a similar approach was not taken in previous studies on mouse sperm, whether this is also the case of epididymal and vas deferens sperm remains unaddressed.

In this work, incubation with Mg^2+^/Ca^2+^ was also seen to activate SCF in ejaculated sperm, also cleaving sperm DNA at TLRs. The sensitivity to Mg^2+^/Ca^2+^, however, was lower compared to Mn^2+^/Ca^2+^. This observation agrees with previous studies where Mg^2+^/Ca^2+^ was detected to elicit SCF in epididymal permeabilized sperm when incubated together with the surrounding epididymal fluid. In mouse, an endogenous nuclease activated in response to the presence of the surrounding fluid added with divalent ions was suggested to evoke SCF; hence, that fluid was hypothesized to be essential to elicit SCF in epididymal and vas deferens sperm [17]. This, nevertheless, does not seem to be the case of ejaculated sperm, as the SCF mechanism was triggered in both permeabilized and non-permeabilized cells. This observation suggests that, at least under the conditions tested here, a transduction pathway stimulated by the activation of a cell-surface receptor that relies upon the intracellular sperm machinery is required for the cleavage of sperm DNA. This does not exclude that the final effector could be an endogenous nuclease, as earlier research reported that such an enzyme could have a vital function in the degradation of internalized exogenous DNA [33]. It is well known that DNAse I enzymes require divalent cations to activate. Interestingly, the separate enzyme subtypes differ on whether they use Mg^2+^ or Mn^2+^ as cofactors [34–36], and these divalent ions exhibit disparities in how the DNA is cleaved. Conversely, monovalent ions such as K^+^ or Na^+^ inhibit the generation of DNA breaks [37]. Related to this, it is worth noting that, in the experiments conducted in the current study, concentration of monovalent ions was the same in all treatments. In addition to the activity of an endogenous nuclease, one should not discard the participation of topoisomerase IIB, as previous investigations indicated that this enzyme is located near to TLRs and it is also involved in cleaving DNA into loop-sized fragments of about 50 Kb [19, 20, 25, 27, 28].

Regardless of whether endogenous DNAse I, topoisomerase IIB or both enzymes are involved in the SCF mechanism, incubation of ejaculated sperm with Mg^2+^/Ca^2+^ or Mn^2+^/Ca^2+^ resulted in an increase of DNA fragments sized between 33 and 194 Kb. Both divalent cations, therefore, appear to switch on a similar mechanism, which leads to the cleavage of DNA at TLRs [19, 20]. As mentioned in the Introduction, the most accepted model of sperm chromatin organization establishes that TLRs are associated to histones rather than to protamines [19, 20]. Thus, in these regions, the DNA is more exposed to nuclease activity and, consequently, more susceptible to become cleaved [7, 17, 24]. The fact that inducing SCF with Mg^2+^/Ca^2+^ or Mn^2+^/Ca^2+^ did not result in an increase in ROS levels supports that, in this case, a non-oxidative mechanism intervenes in the generation of DNA breaks. Indeed, if an oxidative mechanism underlay the activation of SCF, ROS would also cause single-stranded breaks both in protamine-condensed (toroidal structures) and histones-condensed (TLR) regions, thus producing more extensive DNA damage with lower-sized fragments. The results obtained in the current study are therefore compatible with a non-oxidative mechanism, which could be part of the intrinsic apoptotic pathway in sperm [38], designated by some authors as ‘spermptosis’ [39], as DNA fragmentation is one of the features of apoptosis in somatic cells [40–43]. Although previous studies evaluating sperm quality in vitro reported that other apoptotic-like changes are likely to appear together with DNA fragmentation [44, 45], whether this also occurs in ejaculated sperm in vivo has not been addressed. Interestingly, Ribas-Maynou et al. [29] identified that an endogenous nuclease in vasectomized mice is active, suggesting that, in vivo, this enzyme promotes the cleavage of DNA thus rendering it unable to lead to a viable embryo when epididymal storage is excessive. Related to this, it is worth bearing in mind that sperm DNA integrity plays a crucial role during embryo development because, as demonstrated in different species [46–49], it is correlated to embryo development outcomes [46, 50]. Still in agreement with these findings, sperm having undergone SCF are no longer capable of supporting embryonic development when injected into oocytes [27]. All these data point to a limited capability of oocytes to repair paternal DNA breaks, especially those coming from SCF, which drives early embryo development failure and prevents the paternal DNA damage to be passed on the offspring [51].

While earlier research in mice demonstrated that incubation with Mn^2+^/Ca^2+^ and Mg^2+^/Ca^2+^ activates the SCF mechanism in epididymal and vas deferens sperm, the effects of this treatment on sperm function and survival were not previously interrogated. In the present work, neither sperm viability nor ROS generation were affected by incubation with Mn^2+^/Ca^2+^ or Mg^2+^/Ca^2+^. In mammals, sperm DNA fragmentation is usually correlated with oxidative stress, and ROS is understood to be the main source of DNA damage [52–55]. The fact that, even when SCF was induced with Mn^2+^/Ca^2+^ or Mg^2+^/Ca^2+^, ROS levels were low supports that an enzymatic, non-oxidative mechanism would be activated in this case. On the other hand, and in spite of the lack of effect on sperm viability and ROS levels, incubation with Mn^2+^/Ca^2+^ or Mg^2+^/Ca^2+^ decreased motility and increased agglutination. This impact was dose-dependent and was particularly obvious at high concentrations. Previous investigations observed that high Mn^2+^ concentrations in seminal plasma are detrimental to sperm motility [56]. Yet, as the inclusion of 200 μM Mn^2+^ was suggested to exert a protective function during sperm cryopreservation [57], and because in the present study low Mn^2+^/Ca^2+^ concentrations were found to activate SCF, further research should address whether the addition of Mn^2+^ to cryopreservation media can be deleterious for sperm DNA integrity or embryo development [26, 32]. Moreover, the observed increase in sperm agglutination could be attributed to sperm plasma membrane destabilization caused by the interaction between the positive charge of Mn^2+^, Mg^2+^ and Ca^2+^ cations and the negatively charged plasma membrane. Nonetheless, as no effect on the assessment of sperm viability, which evaluates plasma membrane integrity, was observed, more investigations are needed to understand why incubation with Mg^2+^/Ca^2+^ or Mn^2+^/Ca^2+^ leads to sperm agglutination, especially at high concentrations.

## Conclusions

In conclusion, this study demonstrated, for the first time, that Mn^2+^/Ca^2+^ and Mg^2+^/Ca^2+^ are able to induce SCF in ejaculated sperm in vitro. As a result, DNA is cleaved at the TLRs, generating fragments with a length similar to the DNA condensed into toroidal structures, sperm become agglutinated and total motility decreases. The comparison between permeabilized and non-permeabilized sperm suggested that triggering of SCF is related to intracellular components, and flow cytometry data supported that it is independent from oxidative stress. Further research into how the DNA breaks induced by SCF influence fertilization and embryo development is warranted.

## Materials and methods

### Animals and samples

All ejaculates were provided by a local pig farm (Grup Gepork S.L., Masies de Roda, Spain), which follows the ISO certification (ISO-9001:2008) in the production of doses. The Centre follows all the regulations concerning the production and selling of seminal doses (Directive 2010/63/EU; Animal Welfare Law issued by the Regional Government of Catalonia, Spain; and the regulation on Health and Biosafety issued by the Department of Agriculture, Livestock, Food and Fisheries, Regional Government of Catalonia, Spain). As authors did not manipulate any animal but samples were rather provided by the farm, no permission from the local ethics committee was needed.

All seminal samples intended to the experiments defined below came from healthy and sexually mature Pietrain boars (1–3 years old). Ejaculates were collected with the standard hand-gloved method for this species. Immediately after collection, samples were diluted to a final concentration of 33 × 10^6^ sperm/mL in a commercial extender (Vitasem LD, Magapor S.L.; Zaragoza, Spain) and transported at 17 °C to the laboratory.

### Experimental design

Different experiments were designed to test whether the SCF mechanism operates in ejaculated sperm, and to elucidate if it may be triggered in vitro upon incubation with different ions in a dose-dependent manner. In the first and second experiments, whether the activation of the SCF mechanism increases the incidence of DSBs and if these DSBs are located in the TLRs was investigated. The third experiment determined if evoking SCF with Mn^2+^/Ca^2+^ and Mg^2+^/Ca^2+^ has any repercussion on other sperm functional variables, such as motility, viability, agglutination and intracellular levels of reactive oxygen species.

#### Experiment 1: Dose-dependent effect of Mn^2+^/Ca^2+^ and Mg^2+^/Ca^2+^ on the generation of DNA breaks

To assess the generation of DNA breaks after exposure to Mn^2+^ and Mg^2+^ ions, the first experiment incubated ejaculated sperm with these ions, at different concentrations and for different times. In addition, these experiments were conducted in permeabilized and non-permeabilized samples with the aim to address if the SCF mechanism is triggered outside the cell or involves the intracellular machinery. Briefly, three semen doses, each from a separate boar (sperm concentration: 33 × 10^6^ sperm/mL), were pooled and centrifuged at 600 g for 10 min at room temperature to remove preservation medium. Sperm were resuspended in phosphate buffered saline buffer (PBS; 137 mM NaCl, 2.7 mM KCl, 10 mM Na_2_HPO_4_, and 1.8 mM KH_2_PO_4_, pH = 7.5). In the case of permeabilized samples, they were further incubated with 0.25% Triton X-100 for 10 min on ice. Following this, every sample was split into two aliquots (one for Mn^2+^/Ca^2+^ and the other for Mg^2+^/Ca^2+^), and each of these aliquots was in turn divided into 10 tubes of equal volume (five to assess the effects of the dose, and five to determine those of the incubation time). To evaluate the dose–response, samples were incubated with Mn^2+^/Ca^2+^ or Mg^2+^/Ca^2+^ at 0 mM [Control], 0.1 mM, 1 mM, 5 mM or 50 mM (prepared with the appropriate volumes of MnCl_2_, MgCl_2_ and CaCl_2_, all at 0.5 M), for 10 min at 37 °C. To evaluate the time-response, samples were incubated with 10 mM Mn^2+^/Ca^2+^ or Mg^2+^/Ca^2+^ at 37 °C for 0 min (Control), 2 min, 10 min, 30 min or 60 min. After incubation, the incidence of DNA breaks was determined through the Comet assay.

#### Experiment 2: Assessment of the size of the DNA fragments generated by Mn^2+^/Ca^2+^ and Mg^2+^/Ca^2+^ incubations

Pulsed-field Gel Electrophoresis (PFGE) was run to identify the size of the DNA fragments generated after inducing SCF through incubation with Mn^2+^/Ca^2+^ or Mg^2+^/Ca^2+^. Based on the results of experiment 1, 7 mL of non-permeabilized ejaculated sperm from three boars (concentration: 33 × 10^6^ sperm/mL) were centrifuged at 600 g for 10 min at room temperature to remove preservation medium. Then, samples were resuspended in PBS buffer and incubated with 10 mM Mn^2+^/Ca^2+^ or 10 mM Mg^2+^/Ca^2+^ (prepared with the appropriate volumes of MnCl_2_, MgCl_2_ and CaCl_2_, all at 0.5 M) at 37 °C for 30 min. Samples were subsequently subjected to PFGE, as described below, in order to assess the size of the resulting DNA fragments. Negative controls consisted of non-treated sperm in PBS without Mn^2+^/Ca^2+^ or Mg^2+^/Ca^2+^ incubated at 37 °C for 30 min.

#### Experiment 3: Impact of Mn^2+^/Ca^2+^ and Mg^2+^/Ca^2+^ dose and/or incubation time treatments on sperm function and survival

How incubation with different doses of Mn^2+^/Ca^2+^ and Mg^2+^/Ca^2+^ and for different periods affects sperm function and survival was evaluated on the basis of sperm motility with Computer Assisted Sperm Analysis (CASA); sperm agglutination under a phase-contrast microscope; and sperm viability, and levels of total ROS and superoxides through flow cytometry. For all treatments, 50 mL of each sample (N = 3, from three separate boars) were centrifuged at 600 g and room temperature for 10 min to remove the preservation medium. After centrifugation, the supernatant was discarded and the pellet was resuspended in 50 mL of PBS previously tempered at 37 °C. Then, sperm diluted in PBS were distributed in 1-mL aliquots according to the number of treatments and incubation times tested. Following this, the corresponding Mn^2+^/Ca^2+^ or Mg^2+^/Ca^2+^ treatments were added to sperm samples. To evaluate the dose- and incubation time-response, sperm samples were incubated with different concentrations of Mn^2+^/Ca^2+^ or Mg^2+^/Ca^2+^ (Control, 0.1 mM, 1 mM, 5 mM, 10 mM and 50 mM, prepared with the appropriate volumes of MnCl_2_, MgCl_2_ and CaCl_2_, all at 0.5 M) for different incubation times (2 min, 10 min, 30 min and 60 min) at 37 °C. Once done, flow cytometry and CASA analysis were conducted to assess the aforementioned variables.

### Comet assay

The global incidence of sperm DNA breaks was determined with the Comet assay. The protocol used included a previous step for complete chromatin decondensation, which is known to be essential to accurately evaluate DNA fragmentation in pig sperm [58]. First, samples were diluted to 5 × 10^5^ sperm/mL in PBS and mixed 1:2 (v:v) with prewarmed (37 °C) low melting point (LMP) agarose, achieving a final agarose concentration of 0.66% (w:v). Then, 6.5 µL of the mixture was placed onto an agarose-treated slide, which contained a layer of 1% LMP agarose (Thermo Fisher Scientific; Waltham, MA, USA); the drop was covered with an 8-mm diameter round coverslip. After allowing the agarose-sample mixture to solidify for 5 min on the top of a metal cold plate at 4 °C, the coverslip was gently removed. Subsequently, the slide was incubated in three lysis solutions: (1) 0.8 M Tris–HCl, 0.8 M DTT and 1% SDS (pH = 7.5) for 30 min; (2) 0.8 M Tris–HCl, 0.8 M DTT and 1% SDS (pH = 7.5) for 30 min; and (3) 0.4 M Tris–HCl, 0.4 M DTT, 50 mM EDTA, 2 M NaCl, 1% Tween20 and 100 µg/mL Proteinase K (pH = 7.5) for 180 min. Then, DNA was denatured through incubation in an alkaline solution (0.03 M NaOH, 1 M NaCl, pH = 13) at 4 °C for 5 min. Slides were electrophoresed at 1 V/cm for 4 min in an alkaline buffer (0.03 M NaOH, pH = 13). After electrophoresis, samples were incubated in a neutralization solution (0.4 M Tris–HCl, pH = 7.5) for 5 min, and slides were subsequently dehydrated in an increasing ethanol series (70%, 90% and 100% ethanol; 2 min per step). Samples were dried in horizontal position and stored until staining and analysis. For staining, slides were submerged into a solution containing 5 µL of 1 × SYTOX Orange (Invitrogen; Waltham, MA, USA) in 50 mL of distilled water for 15 min.

The prepared Comet samples were visualized under an epifluorescence microscope (Zeiss Imager Z1, Carl Zeiss AG; Oberkochen, Germany) and captured using the Axiovision 4.6 software (Carl Zeiss AG) at a resolution of 1388 × 1040 pixels. At least 100 sperm cells per sample were captured at 100 × magnification, adjusting the exposure time in each capture to avoid overexposure of Comet heads or Comet tails. The quantitative analysis of fluorescence intensity was performed using the automatic function of the CometScore v2.0 software (Rexhoover, www.rexhoover.com). After reviewing the analysis, overlapping comets or signals that did not correspond to comets were eliminated. A minimum of 50 analyzable comets was set as the lowest limit to establish a mean DNA damage intensity, and more pictures were captured and analyzed when this figure was not reached. Data files including Comet head intensity (arbitrary units (AU)), tail intensity (AU), tail length (pixels) and percentage of DNA in the tail (Tail DNA) were exported from CometScore as.csv. To quantify the amount of DNA breaks, the Olive Tail Moment (OTM) was used as a standard parameter and calculated as OTM = (Tail mean intensity − Head mean intensity) × Tail DNA/100.

### Pulsed-field gel electrophoresis (PFGE)

After each treatment, sperm concentration was adjusted to 400 × 10^6^ sperm/mL through centrifugation at 600 g for 5 min and resuspension in PBS. Following this, samples were mixed 1:1 (v:v) with 2% LMP agarose (Thermo Fisher Scientific) previously melted at 38 °C, then poured onto BioRad plug molds (Bio-Rad; Hercules, CA, USA) and finally cooled at 4 °C for 15 min. Thereafter, plugs were unmolded, placed in 2 mL of lysis buffer (10 mM Tris–HCl, 10 mM EDTA, 100 mM NaCl, 20 mM DTT, 2% SDS and 20 mg/mL proteinase K; pH = 8.0), and incubated at 53 °C for 60 min. After incubation, plugs were washed three times in TE buffer (10 mM Tris–HCl, 0.1 mM EDTA; pH = 8).

A half of each plug was cut-off and loaded onto a well of 1% PFGE agarose gel (Pulsed-Field Certified Agarose BioRad; Hercules, CA, USA). A slice of the Low Range PFG DNA Marker (New England Biolabs; Ipswich, MA, USA), which was commercially embedded in agarose, was also loaded. The agarose gel with the standard casting platform frame was placed into a contour-clamped homogeneous electric field apparatus (Bio-Rad CHEF DRIII system) in 0.5 × TBE buffer (Tris–borate 50 mM, EDTA 0.1 mM) at 14 °C. Samples were run at 4 V/cm for 27.1 h with a rotation (angle) of 120° and a pulse change ramp from 6.7 to 33.7 s. Finally, the gel was stained with ethidium bromide and visualized and photographed under ultraviolet light using the GelDoc System (BioRad; Hercules, CA, USA).

For each sample, the intensity of the DNA smear in the gel was quantified employing the Image Studio Lite (LI-COR Biosciences, Lincoln, NE, USA). The DNA ladder was utilized to distinguish the size of DNA fragments as follows: (i) < 33 Kb, corresponding to DNA fragments shorter than a toroid; (ii) between 33 and 194 Kb, corresponding to DNA fragments with sizes compatible with one or multiple toroids; (iii) and > 194 Kb, corresponding to mostly intact DNA (i.e., where SCF did not induce DSBs).

### Sperm motility

Sperm motility was assessed through a CASA system (Integrates Sperm Analysis System, ISAS V1.0; Proiser S.L.; Valencia, Spain) coupled to an Olympus BX41 microscope (Olympus; Tokyo, Japan) with a negative phase contrast field at 100 × (Olympus 10 × 0.30 PLAN objective, Olympus). Semen samples were incubated at 38 °C for 15 min, and 3 µL of each sample was placed into a pre-warmed 20-μm Leja chamber slide (Leja Products BV; Nieuw-Vennep, The Netherlands). Two technical replicates were examined, evaluating 1000 sperm per replicate. Total motility was recorded assuming that a sperm cell was motile when its average path velocity (VAP) was ≥ 10 μm/s.

### Flow cytometry

Flow cytometry analysis were performed using a CytoFLEX flow cytometer (Beckman Coulter, Fullerton, CA, USA), equipped with red, blue and violet lasers (637, 488 and 405 nm). First, sperm concentration was adjusted to 1 × 10^6^ sperm/mL in PBS. Two replicates per sample were examined in each test and three sperm parameters were evaluated: sperm viability, total ROS and superoxides. For this purpose, SYBR-14, 2′7′-dichlorodihydrofuorescein (H_2_DCFDA) and Hydroethidine (HE) fluorochromes were combined with Propidium Iodide (PI) or YO-PRO-1. All fluorochromes were excited with the 488 nm laser. The fluorescence emitted by SYBR-14, YO-PRO-1 and H_2_DCFDA was detected with the FITC channel (525/40), that emitted by HE was collected through the PE channel (585/42), and the fluorescence emitted by PI was detected through the PC5.5 channel (690/50). All fluorochromes were purchased from ThermoFisher (Waltham, MA, USA). Analysis of flow cytometry dot-plots was conducted through the CytExpert Software (Beckman Coulter; Fullerton, CA, USA), and the device was calibrated daily as recommended by the manufacturer.

#### Sperm viability

Sperm viability was determined by staining samples with SYBR-14 (final concentration of 32 nmol/L) and PI (final concentration of 7.5 μmol/L) at 38 °C in the dark for 15 min. The percentages of viable (SYBR-14^+^/PI^−^) and non-viable sperm (SYBR-14^−^/PI^+^ and SYBR-14^+^/PI^+^) were recorded and used for subsequent statistical analyses.

#### *Total ROS levels (H*_*2*_*DCFDA)*

Total ROS levels in sperm were detected through staining with H_2_DCFDA (final concentration of 100 μmol/L), a cell-permeant compound that is oxidized into DCF^+^ in the presence of ROS, and PI (final concentration of 5.6 μmol/L) at 38 °C in the dark for 20 min. After analysis, four subpopulations were identified and recorded: viable sperm with low levels of ROS (DCF^−^/PI^−^); viable sperm with high levels of ROS (DCF^+^/PI^−^); non-viable sperm with low levels of ROS (DCF^−^/PI^+^); and non-viable sperm with high levels of ROS (DCF^+^/PI^+^).

#### Superoxide levels (HE)

Intracellular superoxide levels (O_2_^•−^) were determined after incubation of samples with hydroethidine (HE) (final concentration of 5 µmol/L), an element that is oxidized into E^+^ in the presence of O_2_^•−^, and YO-PRO-1 (final concentration of 31.2 nmol/L) for 20 min at 38 °C in the dark. The fluorescence emitted by E^+^ was detected with the FITC channel and the one emitted by YO-PRO-1 was collected through the PE channel. Spill-over into these two channels was compensated (2.24% and 7.5%, respectively). Four separate subpopulations were identified and recorded: viable sperm with low levels of superoxides (E^−^/YO-PRO-1^−^); viable sperm with high levels of superoxides (E^+^/YO-PRO-1^−^); non-viable sperm with low levels of superoxides (E^−^/YO-PRO-1^+^); and non-viable sperm with high levels of superoxides (E^+^/YO-PRO-1^+^).

### Sperm agglutination

Because sperm became agglutinated after incubation with some treatments, the degree of agglutination was also determined in this study. For this purpose, and following the protocol described by Harayama et al. [59], 250 sperm cells per sample were counted under a phase-contrast microscope at 100 × magnification. Each sperm cell was classified as either agglutinated or non-agglutinated. Two technical replicates per sample were examined.

### Statistical analysis

Statistical analyses were conducted using IBM SPSS for Windows version 27.0 (IBM Corp., Armonk, NY, USA) and GraphPad Prism version 8 (La Jolla, CA, USA). First, normal distribution and homogeneity of variances were examined using Shapiro–Wilk and Levene tests. As parametric assumptions could not be assumed for the data obtained and because samples were paired (repeated measures), a non-parametric two-way ANOVA (Scheirer-Ray-Hare Test) was run. Factors were concentration of Mn^2+^/Ca^2+^ or Mg^2+^/Ca^2+^, and incubation time. Pair-wise comparisons were conducted using the Wilcoxon test. A *P*-value ≤ 0.05 was taken as the limit to consider values statistically significant.

## Data Availability

Data will be shared on reasonable request to the corresponding author.

## Abbreviations

−TX: Non-permeabilized samples
+TX: Permeabilized samples
AU: Arbitrary units
Ca^2+^: Calcium
CASA: Computer assisted sperm analysis
DSB: Double-stranded DNA breaks
H_2_DCFDA: 2′7′-Dichlorodihydrofuorescein
HE: Hydroethidine
LMP: Low melting point
Mg^2+^: Magnesium
Mn^2+^: Manganese
O_2_^•−^: Superoxide ion
OTM: Olive tail moment
PBS: Phosphate buffered saline
PFGE: Pulsed field gel electrophoresis
PI: Propidium iodide
ROS: Reactive oxygen species
SCF: Sperm chromatin fragmentation
TLRs: Toroid-linker regions
VAP: Average path velocity
